# Supplementary material for: Enhancing Radiotherapy Workflow for Head and Neck Cancer with Artificial Intelligence: A Systematic Review
Source: J Pers Med. 2023 Jun 2;13(6):946. doi: 10.3390/jpm13060946 (PMC10301548; doi:10.3390/jpm13060946)
Supplement: Supplementary file 1 [file jpm-13-00946-s001.zip › jpm-2409929-supplementary.pdf]

Table S1. Summary of the studies on the role of artificial intelligence for radiotherapy contouring in head and neck cancer.

Authors	Region of interest	Relevance	Models	Imaging	Metrics	Results	Data
Ibragimov [16] (2017)	OARs	Pilot study	CNN	CT	DSC	DSC varied from 37.4% for chiasm to 89.5% for mandible<br>CNNs showed similar or superior performance for all but submandibular glands and optic chiasm with respect to state-of-the-art AI	50 CT scans
Tam et al. [17] (2018)	OARs	Pilot study	MSVR	CT	DSC	DSC ranged from 66.9% for the left cochlea to 93.8% for the left eye globe	56 HNC
Nikolov et al. [18] (2021)	OARs	External validation (public dataset)	3D U-Net	CT	DSC<br>Surface DSC	No clinical difference between DL model and human	486 HNC (838 scans)
Zhong et al. [19] (2021)	OARs	Dataset size	CNN	CT	DSC	CNN performed best for many OARs (DSC>0.7)	664 HNC
Zhang et al. [20] (2020)	OARs	Time for segmentation	3D encoder–decoder network	CT	DSC<br>HD	Total segmentation time=40.13 s<br>DSC ranged 0.70-0.89 and HD95 ranged 0.7-8.4mm	120 HNC training, 30 validation, 20 test
Brunenberg et al. [21] (2018)	OARs	External validation (different Institution)	2D CNN	CT	DSC<br>HD95	CNN model was a good starting point for delineation of new patients	589 HNC
Chen et al. [22] (2020)	OARs	DL vs AB	3D U-Net	CT	DSC<br>HD<br>HD95<br>MSD	DC performed better than atlas-based (DSC 83%-89% vs 79%-85%) for masticator muscles	56 HNC
Van Dijk et al. [23] (2019)	OARs	DL vs AB	2D CNN	CT	DSC<br>HD<br>∣Δmean-dose∣ and ∣Δmax-dose∣	DL significantly improved segmentation outcomes for most of the 22 OARs compared to AB<br>Adjustment delineation time was slightly reduced for DL (36±7 vs 59±14min)	549 HCN training, 40 validation, 104 test

Urago et al. [24] (2021)	OARs	DL vs AB	U-Net	CT	DSC<br>HD<br>MDA	No significant difference between DL and AB, considering DSC, HD, and MDA	30 HNC
Guo et al. [25] (2021)	OARs	DL vs AB	2D U-Net and 3D U-Net	CT	HD<br>DSC<br>MDA<br>Jaccard index<br>ΔDose	No significant dose-volume measurements differences for DL vs manual contouring<br>No correlations between topological indices and dosimetric difference	10 nasopharyngeal carcinoma
Kim et al. [26] (2021)	OARs	DL vs DIR	Fully Convolutional DenseNet	CT	DSC<br>HD<br>MSD	DLSm achieved better performance than both DLSu and DIR (DSC 0.83 vs. 0.80 vs. 0.70), mainly for glandular structures	100 HNC
Brouwer et al. [27] (2020)	OARs	Manual adjustments	2D CNN	CT	Median adjustment	Low median adjustment (<2 mm) for all OARs<br>Some structures needed quite high local adjustments<br>The DL model usually under-estimated the segmentation area	103 HNC
Oktay et al. [28] (2020)	OARs	Manual adjustments	3D U-Net	CT	DSC<br>Working time	Correction time of segmented OARs was 4.98 (95% CI, 4.4-5.5) min/scan, corresponding to a mean 93% reduction in time in comparison to manual contouring	242 HNC
Bai et al. [29] (2022)	OARs	Manual adjustments	U-Net	CT	DSC<br>HD95	AI–assisted contour editing improved initial DSC (>10%) and HD95 (reduced almost by half)<br>Processing times were ~20 ms for each contour update	58 HNC
Wong et al. [30] (2021)	OARs	Manual adjustments	U-Net	CT	degree of edits required<br>overall satisfaction	The majority of OARs required minimal edits (mean subjective editing score≤2/5; mean DSC and 95% HD was≥0.90 and≤2.0 mm)<br>Mean OAR satisfaction score was 4.4/5.0	54 HNC
Liu et al. [31] (2020)	OARs	Small volumes	CLAF-CNN	CT	DSC	CLAF-CNN outperformed state-of -the-art attention-based segmentation methods in OAR segmentation task, with average DSC of 0.80	50 HNC
Liu et al. [32] (2021)	OARs	Multi-view images	CNN	CT	DSC<br>HD	Residual improvement for a multi-view (coronal, sagittal, and transverse plane)	220 HNC

						segmentation model vs. standard 2D model was observed (DSC: 0.83 vs. 0.86)	
Iyer et al. [33] (2021)	OARs	Multi-view images	2.5D CNN	CT	DSC<br>HD95	Multi-view ensemble model was found to avoid coarse segmentation errors compared to single-view models	242 HNC
Wong et al. [34] (2020)	OARs	Multi-view images	Deep convolutional neural network models (one model per structure) based on a U-net architecture	CT	DSC<br>HD95	DL results for spinal cord, parotid gland, submandibular glands were comparable to expert human inter-observer variability<br>DC-EC contours were less similar than EC-EC contours for the neck CTV (DSC: 0.72 vs 0.79; HD95: 10.93 vs 6.75 mm)	10 HNC (53 structures)
Zhang et al. [35] (2021)	OARs	Multi-view images	WAU-net	CT	DSC<br>HD95<br>MSD	WAU-net showed similar results to state-of-the-art methods, outperforming in 3 out of 10 OARs	115 HNC
Tong et al. [36] (2018)	OARs	Multiple networks	Fully convolutional neural network constrained by a SRM	CT	DSC<br>ASD<br>95%SD	SRM significantly improved segmentation accuracy for 9 organs, showing better performance than FCNN alone	32 HNC
Liang et al. [37] (2018)	OARs	Multiple networks	ODS-Net	CT	DSC	ODS-Net provided significant higher DSC than FCNN in 10 out of 11 OARs	208 nasopharyngeal carcinoma
Men et al. [38] (2019)	OARs	Multiple networks	CNN Cascades:<br>SRD and FSU<br>U-Net	CT	DSC<br>HD	CNN Cascades achieved best performance with mean DSC of 0.90 (SRD: 0.86, FSU: 0.87, and U-Net: 0.85) and mean HD of 3.0 mm (SRD: 4.0, FSU: 3.6, and U-Net: 4.4)<br>Mean segmentation time per patient for FSU, U-Net and CNN Cascades was, respectively, 10.6, 5.8, and 5.5 min	100 HNC
Zhong et al. [39] (2019)	OARs	Multiple networks	Boosting ResNet	CT	DSC<br>HD95<br>VOE%	Boosting-based cascaded CNN showed a higher capability in segmentation than U-Net and FCNN	140 nasopharyngeal carcinoma
Tappeiner et al. [40] (2019)	OARs	Multiple networks	HighRes3DNets	CT	DSC<br>HD95	The coarse stage achieved a DSC of 0.71±0.19 and HD95 of 7.5±22.9 mm<br>In the fine stage the overall segmentation results improved to DSC=0.72±0.18 and HD95=6.3±16.2 mm	40 HNC

Sultana et al. [41] (2020)	OARs	Multiple networks	3D U-Net combined with a generative adversarial network	CT	DSC<br>MSD<br>HD95	Hierarchical U-Net-GAN achieved better segmentation performance compared to U-Net-GAN and U-Net alone, with a DSC of 0.87 for parotid and submandibular glands	20 HNC
Hänsch et al. [42] (2019)	OARs	Multiple networks	2D U-Net<br>ensemble 2D U-Net<br>3D U-Net	CT	DSC	No significant differences in the median DSC were observed between the proposed models	254 HNC
Tappeiner et al. [43] (2020)	OARs	Dataset size	3D CNN	CT	DSC<br>HD95<br>SD	Twelve images were sufficient for accurate auto-segmentation, with only a 3% decrease of DSC for OARs compared to a series of 25 images	25 HNC
Fang et al. [44] (2021)	OARs	Dataset size	U-Net	CT	DSC	Compared to the best performance, optic nerves and lenses reached 95% of their best effect at 200 patients, while the other organs reached 95% of their best effect at 40 patients	1160 HNC
Hague et al. [45] (2021)	OARs	MRI-based models	U-Net	CT<br>MRI	DSC<br>MDA	For parotid and submandibular glands, the MRI-based model achieved better results compared to the CT-based model<br>Performances were affected by the MRI sequence	621 HNC
Dai et al. [46] (2021)	OARs	MRI-based models	R-CNN	MRI	DSC<br>HD95<br>MSD	R-CNN using MRI sequences achieved a mean DSC of 0.78, outperforming the same model without recognition substructures (DSC of 0.73)	60 HNC
Korte et al. [47] (2021)	OARs	MRI-based models	CNN-based auto-segmentation	MRI	DSC<br>MSD	CNNs were suitable to auto-segment the parotid and submandibular glands on MRI images	31 HNC
Dai et al. [48] (2021)	OARs	Multi-modality	Cycle-GAN	CT<br>sMRI	DSC<br>HD95<br>MSD	The proposed model achieved (mean [range]):<br>- DSC: 0.77 [0.58, 0.90]<br>- HD95: 2.9 mm [1.3, 7.6] mm<br>- MSD: 0.9 mm [0.4, 1.8] mm<br>- RMS: 1.4 mm [0.7, 3.2] mm	70 HNC
Kieselmann et al. [49] (2022)	OARs	Multi-modality	2D CNN	CT<br>MRI	DSC<br>HD<br>MSD	A CNN could be trained using high-quality synthetic MR images<br>The proposed technique may be valuable in case of non-annotated images	202 CT scans and 27 MRI

Comelli et al. [50] (2020)	GTV (primary)	Multi-modality	3D CNN	PET	DSC<br>HD<br>MHD	DSC>0.88<br>HD<1.5 voxel<br>Mahalanobis distance<0.8 voxel	25 HNC
Naser et al. [51] (2020)	GTV (primary)	Multi-modality	2D and 3D U-Net	CT<br>PET	DSC	Minimal (but significant – p-value=0.04) mean DSC improvement for the 3D model (0.69) vs 2D model (0.67).	201 HNC
Groendahl et al. [52] (2021)	GTV (primary)	Multi-modality	2D U-Net	CT<br>PET	DSC	PET/CT-based CNN model showed the best segmentation performance (DSC=0.74) compared to PET threshold method (0.62), and CT (0.66).	197 HNC
Guo et al. [53] (2019)	GTV (primary)	Multi-modality	Dense-Net<br>3D U-Net<br>Dense-Net	CT<br>PET	DSC<br>MSD<br>HD95<br>DMC	Multi-modality Dense-Net obtained better results than 3D U-Net or single-modality input Dense-Net, with DSC=0.73, MSD=3.1mm, HD95=9.0 mm and DMC=4.8 mm using both PET and CT input images<br>On large GTV size (>30cc) the model produced better predictions than on smaller target volumes	140 HNC training, 35 validation, 75 test
Gurney-Champion et al. [54] (2020)	GTV (primary)	MRI-based models	3D U-Net	MRI	DSC,<br>ΔADC	DSC=0.87<br>ΔADC=1.9%<br>The proposed network performed worse on patients receiving induction-chemotherapy	48 HNC, 3 MRI-Linac
Ren et al. [55] (2021)	GTV (primary)	Multi-modality	3D U-Net	CT<br>MRI<br>PET	DSC<br>HD95<br>MSD	Training on combined CT-PET-MRI provided limited improvement over CT-PET alone, which represents the best bimodal training	153 HNC
Moe et al. [56] (2021)	GTV (primary)	Multi-modality	U-Net	CT<br>PET	DSC<br>HD95<br>MSD	CT-based, PET-based, and PET/CT-based models for GTV contouring obtained a mean DSC of 0.55, 0.69, and 0.71, respectively<br>Models based on PET/CT images identified 86% of the true GTV structures vs 55% for CT-based models	197 HNC
van der Veen et al. [57] (2020)	GTV (lymph node)	Observer variability	3D CNN	CT	MSD<br>DSC<br>HD95	DSC ranged from 46% (level Vc) to 82% (level II-IVa)<br>Automatic delineation required 8 vs 15 minutes of manual contour, and showed a lower inter-observer variability	69 HNC

van der Veen et al. [58] (2019)	OARs	Observer variability	3D CNN	CT	DSC ASSD	Inter-observer variability for manually corrected auto-contours was smaller than intra-observer variability The time needed to fix auto-delineations was significantly shorter than for manual delineations (23 vs 34 min)	15 HNC

Abbreviations: AB=Atlas Based. 95%SD=95% Maximum Surface Distance. ASD=Average Surface Distance. ASSD=Average Symmetric Surface Distance. CLAF-CNN=Cross-Layer Attention Fusion Network. CNN=Convolutional Neural Network. Cycle-GAN=Cycle-Consistent Generative Adversarial Network. DC=Deep learning-based auto-segmented Contours. DIR=Deformable Image Registration. DLSm= matched patients in the deep learning test set. DL=Deep Learning. DLSu=unmatched patients in the deep learning test set. DMC=Displacement of Mass Centroid. DSC=Dice Similarity Coefficient. EC=Expert radiation oncologist Contour. FCNN =Fully Convolutional Neural Network. FSU=Fine Segmentation Unit. GTV=Gross Tumor Volume. HD95=95th percentile Hausdorff Distance. HighRes3DNets=High-Resolution 3D Network. HNC=Head and Neck Cancer. MDA=Mean Distance to Agreement. MRI-Linac=Magnetic Resonance Imaging Guided Linear Accelerator. MSD=Mean Surface Distance. MSVR=Multi-output Support Vector Regression. OAR=Organ at Risk. ODS-Net=Organs at risk Detection and Segmentation Network. R-CNN=Regional Convolutional Neural Network. RMS=Residual Mean Square Distance. RO=Radiation Oncologist. SD=Surface Dice score. sMRI=Synthetic Magnetic Resonance Imaging. SRD=Simple Region Detector. SRM=Shape Representation Model. U-Net-GAN=U-Net Based Discriminator for Generative Adversarial Network. VOE%=Volumetric Overlap Error. WAU-net=Weaving Attention U-net. ΔADC=Absolute percentage change in Apparent Diffusion Coefficient.

Table S2. Summary of the studies on the role of artificial intelligence for radiotherapy planning optimization in head and neck cancer.

**Authors**	**Models**	**Metrics**	**Results**	**Data**
McIntosh et al. [6] (2017)	cARF	Several D% of PTVs and OARs Conformation Number (Van't Riet)	Automated vs. clinical plans evaluation criteria: - average 0.6% higher dose for target coverage - average 2.4% lower dose at the OARs - no statistically significant difference in the conformation number	66 oropharynx HNC patients 54 training, 12 test 59 IMRT, 7 VMAT
Fan et al. [63] (2019)	ResNet	Average deviation between predicted and TPS calculated dose of several D% and V30, for PTVs and OARs	No difference between prediction and real clinical plans for all clinically relevant DVH indices, except brainstem, right and left lens No differences between the automatically generated plans and the predicted plans except for PTV70.4	270 IMRT plans 195 training, 25 validation, 50 test

Nguyen et al. [64] (2019)	HD U-Net	D95, D98, D99, Dmax of PTV<br>Homogeneity Conformation Number (Van't Riet), Dmean, Dmax of all structures	HD U-Net performed better than the standard U-net and DenseNet for all metrics<br>Predicted OAR Dmax within 6.3% and Dmean within 5.1% of the prescription dose on the test data	120 HNC patients (VMAT)<br>80 train, 20 validation (5-fold cross validation), 20 test
Miki et al. [65] (2020)	HD U-Net	HI, Dmax, Dmean, D2cc	HD U-Net achieved more accurate dose predictions to the actual dose of clinical plans than the mFBP method	81 HNC patients with oropharynx or hypopharynx tumors (VMAT)
Li et al. [66] (2021)	cGAN	Several D% of OARs, CI, HI	Comparison between manual and AI plans:<br>- no difference in Dmean of left/right parotid and oral cavity<br>- no differences in Dmax at 0.01cc of brainstem and cord + 5mm margin<br>- Body Dmax higher than TPS plans	231 oropharyngeal IMRT<br>200 training, 16 validation, 15 test
Gronberg et al. [67] (2021)	3D-DDU-Net	Average MAE of dose distributions;<br>D1, D95, and D99 for the high-, medium-, and low-risk targets; D0.1cc and Dmean for each OAR	Average MAE=2.56 Gy on the test set<br>Predicted target DVH metrics within 3% of the clinical plans on average<br>Predicted OAR DVH metrics within 2 Gy of the clinical plans on average	340 HNC (IMRT)<br>200 training, 40 validation, 100 test
Sher et al. [68] (2021)	Commercially-available DST (QuickMatch, Siris Medical)	Comparison of OAR "dose directive" between physician (PD) and AI (AD)	Clinical plans achieved mean dose reductions between 4.3 to 16 Gy with PD, and 5.6 to 9.1 Gy with AD<br>HD reduced OAR dose objectives >3 Gy in 22% to 75% of cases	276 HNC (VMAT) for training<br>50 as test
Carlson et al. [69] (2016)	Single and multiple linear regression; Random forest; Cubist	MAE and RMSE between predicted and delivered MLC positions	H&N plans with 1%/2 mm gamma criteria had an average increase in passing rate of 4.17% (SD=1.54%)	74 VMAT plans from 3 institutions, of which 41 HNC
Koike et al. [70] (2020)	2D Cycle-GAN	Artifact Index for image quality evaluation;<br>Several D% of DAH of the oral cavity between uncorrected/automatic and water override	Artifact Index (automatic vs. uncorrected)=13.2±4.3, 267.3±113.7<br>Greater dose differences between reference water plan and uncorrected, than automatic correction	15 HNC patients (IMRT) used as test set, 92 used in train/validation split from unpaired domains (w/ and w/o artifacts)
Scholey et al. [71] (2022)	3D U-Net	MAE between pairs of MVCT and sMVCT (generated from MRI)<br>Several D% of PTVs and OARs for 4 representative VMAT plans in test set	sMVCT vs. MVCT:<br>- MAE were 93.3±27.5, 78.2±27.5, and 138.0±43.4 HU for whole body, soft tissue, and bone volumes, respectively	120 HNC<br>96 training, 6 validation, 18 test

			- dose differences within 2Gy<br>- average passing rate of 98.9±1.0% and 96.8±2.6% at 3%/3 mm and 2%/2 mm criteria, respectively	

Abbreviations: AD=Artificial Directive. cGAN=Conditional Generative Adversarial Network. CI=Conformity Index. cARF=Contextual atlas regression forest. DAH=Dose Area Histogram. DST=Decision Support Tool. HD=Hybrid Directive. HD U-Net=Hierarchically Densely Connected U-Net. HI=Homogeneity Index. HU=Hounsfield Unit. IMRT=Intensity Modulated Radiation Therapy. MAE=Mean Absolute Error. mFBP=Modified Filtered Back Projection. MRI=Magnetic Resonance Imaging. PD=Physician Directive. ResNet=Residual Network. RMSE=Root Mean Squared Error. SD=Standard Deviation. sMVCT=synthetic Megavoltage Computed Tomography. VMAT=Volumetric Modulated Arc Therapy. 2D Cycle-GAN=2D Cycle-Consistent Generative Adversarial Network. 3D-DDU-Net=3D dense dilated U-Net.

Table S3. Summary of the studies on the role of artificial intelligence for radiotherapy delivery in head and neck cancer.

Authors	Models	Metrics	Results	Data
Maspero et al. [72] (2020)	2D Cycle-GAN (three models: one per anatomical site, and one with all sites)	Image similarity: MAE/ME between rCT and: sCT, CBCT, planning CT<br>Voxel-wise relative dose differences in high dose regions (>90% of the prescribed dose) between rCT and: sCT, planning CT<br>Gamma analysis at 3%/3 mm and 2%/2 mm relative to dose on rCT for regions with dose >10% of the prescription dose	The models' MAEs were compatible in terms of range and with average values within one SD<br>Similarity between sCT and rCT was higher than between CBCT and rCT<br>Mean dose differences <0.5% in high-dose regions<br>H&N average pass rates at 3%/3 mm: sCT vs rCT 99.3±0.4; CT vs rCT 98.7±1)	33 HNC<br>15 training, 8 validation, 10 test
Barateau et al. [73] (2020)	Standard GAN	Comparison of pCT with CTref with respect to:<br>- image endpoints: MAE and ME of HU<br>- dosimetric endpoints: MAE between DVHs<br>3D gamma analysis (2%/2mm)	Image endpoints MAEs and MEs:<br>- DLM: 82.4 and 17.1 HU<br>- HU-D curve method: 266.6 and 208.9 HU<br>- DAM: 113.2 and 14.2 HU<br>- DIR: 95.5 and −36.6 HU<br><br>MAE from DLM significantly lower than all other methods<br>No significant differences in parotid Dmean between DLM and other methods	44 HNC (VMAT)

			Significant differences between DLM and other methods for the 3D gamma analysis. DIR had best gamma results: 98.8±0.7%	
Gan et al. [74] (2021)	Workflow Box 2.0, DLCExpertTM, Mirada Medical Ltd., UK	Dmean and NTCP variation between original and rCT plan for manual and automatic segmentation (15 OARs on 15 rCTs)	Average ∣Dmean∣ variation of PGs:<br>- HS (3 observers): 1.40 Gy<br>- DIR: 3.64 Gy<br>- DLC: 3.72 Gy<br>DLC had highest ∣Dmean∣ variation (5.13 Gy) in middle PCM<br><br>90th percentile ∣NTCP∣ variation (135 models per 15 pz =2025 results):<br>- DIR: 2.19%<br>- DLC: 2.24%<br>- HS: 1.10%<br>- SAS: 1.50%	15 HNC (IMRT or VMAT)
Chen et al. [75] (2021)	2D U-Net	Image quality: HU accuracy, SNR, SSIM<br><br>OARs contours between eCBCT/oCBCT and rCT:<br>Quantitative: mean DSC, HD, COM displacement<br>Qualitative: visual scoring	eCBCT OARs had significant improvement on mean pixel values in terms of SNR and SSIM<br><br>eCBCT-to-rCT vs. oCBCT-to-rCT (enhanced always better):<br>- DSC: 0.83±0.06 vs. 0.70±0.13<br>- HD: 0.42±0.13 cm vs. 0.72±0.25 cm<br>- COM: 0.28±0.19 cm vs. 0.44±0.22 cm<br><br>Visual scoring showed that OAR segmentation was more accessible on eCBCT than oCBCT images	train: 40 HNC patients (CT + first fraction CBCT)<br>test: 15 HNC patients (rCT + oCBCT)
Ma et al. [76] (2022)	U-Net	DSC, HD95, and ASD on 7 OARs: mandible, left and right parotid glands, left and right SMGs, and left and right masseters	The best contours were generated using DIR as image registration algorithm, with mean DSC=86.5, mean HD95=2.54 mm, and mean ASD=0.59 mm	37 HNC patients
Liang et al. [77] (2022)	TTO method applied to:<br>CNN<br>FAIM<br>VoxelMorph<br>VTN	DSC, HD95 on 17 OARs	DSC and HD95 always improved when applying TTO: average maximum improvement of 0.04 (5%) for DSC and 0.98 mm (25%) for HD95	239 HNC patients with squamous cell carcinoma

Guidi et al. [78] (2016)	Cluster analysis: K-means; SVM	Organ warping trend during RT obtained from volume and dose variations of parotid glands during the 6 weeks of therapy	No re-planning needed for an average of 86.7% of cases in the first three weeks<br>During the last 2 weeks, a mean of 23.1% of cases were classified as "correct treatment", 59.3% as “need re-planning”, 11.8% of cases were affected by biases, and 5.9% generated a warning	90 HNC patients<br>41 training, 49 test
Harms et al. [79] (2020)	2D Cycle-GAN	Differences between the CT-based and CBCT-based RSP maps in terms of MAE, ME, PSNR, SSIM<br>Proposed method compared to DIR and two other DL methods	RSP CT-based vs. CBCT-based:<br>- MAE: 0.06±0.01<br>- ME: 0.01±0.01<br>The proposed method statistically outperformed the benchmark DL methods	23 HNC
Lalonde et al. [80] (2020)	2D U-Net	MAE and ME in HU between uncorrected/scatter-free and scatter-corrected images<br>RMSE of proton ranges between reference CT and corrected CBCT<br>2%/2 mm gamma analysis	ME:<br>- uncorrected/scatter-free: -28.6 HU<br>- scatter-free/scatter-corrected: -0.8 HU<br>MAE:<br>- uncorrected/scatter-free: 69.6 HU<br>- scatter-free/scatter-corrected: 13.4 HU<br>RMSE proton ranges: 0.73 cm<br>Gamma pass rate of 98.89% at 2%/2 mm for plans optimized on scatter free images and re-calculated on scatter-corrected images (10 pz)	48 HNC (VMAT)<br>29 training, 9 validation, 10 test<br>Training was done on MonteCarlo simulated CBCT projections

Abbreviations: aCT=adaptive planning CT. ASD=Average Surface Distance. CNN=Convolutional Neural Network. COM=Center of Mass. CTref=reference CT. DAM=Density Assignment Method. DIR=Deformable Image Registration. DLC=Deep Learning Contouring. DLM=Deep Learning Model. DLSm=Deep Learning Segmentation matched. DLSu=Deep Learning Segmentation unmatched. DSC=Dice Similarity Coefficient. eCBCT=enhanced CBCT. FC-DenseNet=Modified fully convolutional DenseNet. FND=False-negative DSC. FPD=False-positive DSC. GAN=Generative Adversarial Network. HD=Hausdorff distance. HD95=95th percentile Hausdorff distance. HS=Human Segmentation. HU=Hounsfield Unit. HU-D=Hounsfield Unit Density. MAE=Mean Absolute Error. ME=Mean Error. MSD=Mean Surface Distance. oCBCT=On the same day CBCT. pCT=pseudo-CT. PCM=Pharyngeal Constrictor Muscle. PG=Parotid Gland. PSNR=Peak Signal-to-Noise Ratio. rCT=repeat-CT. RgDL=registration-guided DL. RMSE=Root Mean Squared Error. RSP=Relative Stopping Power. SAS=Semi Auto-Segmentation. SD=Standard Deviation. SMGs=Submandibular Glands. SNR=Signal-To-Noise-Ratio. SSIM=Structural Similarity Index Measure. SVM=Support Vector Machines. TTO=test-time optimization. VTN=Volume Tweening Network. 2D Cycle-GAN=2D Cycle-Consistent Generative Adversarial Network.
